# Supplementary material for: Nonpharmacological pain relief for labour pain: knowledge, attitude, and barriers among obstetric care providers
Source: PeerJ. 2024 Feb 2;12:e16862. doi: 10.7717/peerj.16862 (PMC10840495; doi:10.7717/peerj.16862)
Supplement: Supplemental Information 1 [file peerj-12-16862-s001.docx]

**Questionnaire to assess** **Utilization of Nonpharmacological Pain Relive Methods for Maternity Care among Health Care Providers at Maternal and Child Hospital in Najran KAS**

**Informed consent**

**Researchers: Dr: Wafaa Taha Elgzar, Dr: Majed Elshahrani and Dr: Heba Abdelfatah Ibrahim**

**Project number:** NU/DRP/MRC/12/2

Hello, we are a group of researchers from Najran University, and we are currently conducting a research project entitled " **Non-pharmacological Labour Pain Relive Knowledge, Attitude, and Barriers among Obstetric Care Providers in Najran, Saudi Arabia: A Cross-Sectional Study"**

Presenting to you the research questionnaire, knowing that the data will be treated in strict confidentiality; No personal identification will be required. Your rejection to participate will not have any penalties at any time. Your participation is appreciated, and it's the key to study success. Also, you can see the study results after publication. Can you fill out the related questionnaire?

Yes ( ) No ( )

For any queries, please contact: Dr: **Wafaa Taha Elgzar**

Telephone: 00966545969866

Email: wtelgzar@nu.edu.sa

**Non-pharmacological Labour Pain Relive Knowledge, Attitude, and Barriers among Obstetric Care Providers in Najran, Saudi Arabia: A Cross-Sectional Study"**

**A. Demographic variables**

**Age:**

**Sex** 1. Male ( ) 2. Female ( )

**Nationality** 1. Saudi ( ) 2.Egyptian ( )

3. Sudanese ( ) 4. Filipino ( )

5. other, specify:

5. Indian ( ) 5. Others ( )

**Religion** 1. Muslim ( ) 2. Cristian ( )

3. Hindu religion ( ) 4. Others ( )

**Marital status** 1. Single ( ) 2. Married ( )

3. Divorced ( ) 4. Widowed ( )

**Educational level**: 1. High diploma ( ) 2. Bachelor's degree ( )

3. Master's degree ( ) 4. Ph.D. ( )

**Monthly income** 1. Less than 5000 SAR ( ) 2. 5000-10000 SAR

3. More than 10000 SAR ( )

**B. Work-related data :**

**Profession**  1. Physician ( ) 2. Nurse ( )

3. midwife ( )

**Years of experience**:……………………………………………………………………………………

**Providers: patient ratio** 1. 1:4 ( ) 2. 1: 6 ( )

3. 1: 8 ( ) 4. Undetermined ( )

**Working hours:** 1. 8 hours ( ) 2. 12 hours ( )

3. More than 12 hours ( )

**Is there a guideline for using nonpharmacological pain relief in your unit?**

1. yes ( ) 2. No ( )

**Have you received any training related to the utilization of nonpharmacological pain relief before?**

1. Never received ( ) 2. Yes, during my formal education ( )

3. yes, during my postgraduate education 4. Yes, training session after employment.

**C. Knowledge related to Nonpharmacological pain relief: Please answer the flowing yes or no questions regarding Nonpharmacological pain relief.**

| **Question** | **True** | **False** |
| --- | --- | --- |
| 1. **Nonpharmacological pain relief is an intervention that does not involve using medications to treat pain.** |  |  |
| 1. **Using nonpharmacological pain relief requires patient consent.** |  |  |
| 1. **Nonpharmacological pain relief methods have a physiological background in the body.** |  |  |
| 1. **Some nonpharmacological pain relief methods can help release of endorphins.** |  |  |
| 1. **Some nonpharmacological pain relief used to divert penitent attention from pain.** |  |  |
| 1. **Some nonpharmacological pain close pain gate.** |  |  |
| 1. **What are the main types of nonpharmacological pain relief (select all the right answers)** | | |
| 1. Co-cognitive-behavioral | | |
| - Guided imagery | | |
| - Relaxation | | |
| - Positive reinforcement | | |
| - Distraction | | |
| - Virtual reality | | |
| 1. Physical | | |
| - Thermal stimulation (cold or hot) | | |
| - Trans electrical nerve stimulation | | |
| - Message | | |
| - Breathing technique | | |
| - Positioning | | |
| - Hydrotherapy/ patient bathing | | |
| - Resting | | |
| - Acupuncture/acupressure | | |
| - Herbal drink | | |
| 1. Emotional | | |
| - Therapeutic touch | | |
| - Therapeutic communication | | |
| 1. Environmental comfort | | |
| 1. Patient-family involvement | | |
| 1. **What are the benefits of nonpharmacological pain relief during labour (select all the right answers)** | | |
| 1. Have lower side effects than medication | | |
| 1. Lower cost | | |
| 1. More available | | |
| 1. Patient-centered | | |
| 1. Building trust in the therapeutic relationship | | |
| 1. It can be used at home. | | |
| 1. More relaxing | | |
| 1. More available | | |

**D. Attitude toward nonpharmacological method utilization for pain relief.**

| Statement | Strongly agree | Agree | Neutral | Disagree | Strongly disagree |
| --- | --- | --- | --- | --- | --- |
| **I think that nonpharmacological pain relief methods are** | | | | | |
| 1. the utilization of nonpharmacological pain relief can make pain management more available. |  |  |  |  |  |
| 1. the utilization of nonpharmacological pain relief could reduce the pain management cost. |  |  |  |  |  |
| 1. Nonpharmacological pain relief can be applied in different health care setting. |  |  |  |  |  |
| 1. Nonpharmacological pain relief is concerned with patient comfort. |  |  |  |  |  |
| 1. Nonpharmacological pain relief can help in building trustful relationship between patient and health care providers. |  |  |  |  |  |
| 1. Nonpharmacological pain relief is more suitable for home utilization. |  |  |  |  |  |
| 1. I could delay pharmacological pain relief to tray nonpharmacological methods. |  |  |  |  |  |
| 1. I will recommend nonpharmacological pain relief for my patients. |  |  |  |  |  |
| 1. Think nonpharmacological methods are necessary for managing pain* |  |  |  |  |  |
| 1. The belief that you have a responsibility and obligation to manage pain* |  |  |  |  |  |

**E. barriers to Nonpharmacological pain relief utilization**

| Barriers for utilization | Strongly agree | Agree | Neutral | Disagree | Strongly disagree |
| --- | --- | --- | --- | --- | --- |
| 1. Healthcare providers' strong belief in pharmacological methods |  |  |  |  |  |
| 1. Patient unwilling |  |  |  |  |  |
| 1. Patient health beliefs |  |  |  |  |  |
| 1. Lack of knowledge |  |  |  |  |  |
| 1. Inadequate training to use |  |  |  |  |  |
| 1. Lack of time |  |  |  |  |  |
| 1. Pain too sever |  |  |  |  |  |
| 1. Policy restriction |  |  |  |  |  |
| 1. Work loud |  |  |  |  |  |
| 1. Lack of equipment |  |  |  |  |  |
| 1. Insufficient motivation |  |  |  |  |  |
| 1. Cultural differences between patients and healthcare providers |  |  |  |  |  |

Thanks for your participation.
